# Optimizing Column Length and Particle Size in Preparative Batch Chromatography Using Enantiomeric Separations of Omeprazole and Etiracetam as Models: Feasibility of Taguchi Empirical Optimization

**DOI:** 10.1007/s10337-018-3519-z

**Published:** 2018-04-25

**Authors:** Jörgen Samuelsson, Marek Leśko, Martin Enmark, Joakim Högblom, Anders Karlsson, Krzysztof Kaczmarski

**Affiliations:** 1 Department of Engineering and Chemical Sciences, Karlstad University, 651 88 Karlstad, Sweden; 2 Department of Chemical Engineering, Rzeszow University of Technology, 35 959 Rzeszow, Poland; 3 Akzo Nobel Pulp and Performance Chemicals AB, 445 80 Bohus, Sweden; 4 AstraZeneca R&D, 431 83 Mölndal, Sweden

**Keywords:** Preparative chromatography, Omeprazole, Etiracetam, Optimization of productivity, Taguchi optimization, Equilibrium–dispersive model

## Abstract

The overreaching purpose of this study is to evaluate new approaches for determining the optimal operational and column conditions in chromatography laboratories, i.e., how best to select a packing material of *proper particle size* and how to determine the *proper length* of the column bed after selecting particle size. As model compounds, we chose two chiral drugs for preparative separation: omeprazole and etiracetam. In each case, two maximum allowed pressure drops were assumed: 80 and 200 bar. The processes were numerically optimized (mechanistic modeling) with a general rate model using a global optimization method. The numerical predictions were experimentally verified at both analytical and pilot scales. The lower allowed pressure drop represents the use of standard equipment, while the higher allowed drop represents more modern equipment. For both compounds, maximum productivity was achieved using short columns packed with small-particle size packing materials. Increasing the allowed backpressure in the separation leads to an increased productivity and reduced solvent consumption. As advanced numerical calculations might not be available in the laboratory, we also investigated a statistically based approach, i.e., the Taguchi method (empirical modeling), for finding the optimal decision variables and compared it with advanced mechanistic modeling. The Taguchi method predicted that shorter columns packed with smaller particles would be preferred over longer columns packed with larger particles. We conclude that the simpler optimization tool, i.e., the Taguchi method, can be used to obtain “good enough” preparative separations, though for accurate processes, optimization, and to determine optimal operational conditions, classical numerical optimization is still necessary.

**Electronic supplementary material:**

The online version of this article (10.1007/s10337-018-3519-z) contains supplementary material, which is available to authorized users.

## Introduction

Preparative chromatography is extensively used for peptides, bio separations, chiral separations, as well as the new biosimilar drugs such as mRNA and oligonucleotides [1–7]. Batch-mode process chromatography is the best generic method to obtain pure drug/drug candidate components in amounts under 10 kg in the discovery stage of pharmaceutical development. The process is mainly carried out using empirical optimization methods, such as touching-band separation, but also using numerical optimization based on chromatographic models of varying complexity [6, 8–14]. It is of highest importance for both preparative and analytical chromatography to take a scientific step from using empirical data and instead use powerful modeling to secure generation of scientific and mechanistic understanding (predictive science). This is well in line with the upcoming and for the pharmaceutical industry important ICHQ12 guideline [15]. There are still no strict criteria for successful preparative separation, despite some longstanding rules of thumb, such as having a retention factor as small as possible for the first-eluted component collected and dissolving the sample in a concentration close to its solubility in a particular eluent [6, 16].

One recent numerical study based on the chiral resolution of racemic omeprazole on an amylose tris (3,5-dimethyl phenyl carbamate)-coated macroporous silica column investigated how maximum productivity depends on the maximum allowed pressure drop and on the packing material particle size for fixed-size analytical columns [17]. It was found that the optimum particle size was large for separations conducted at low pressures, but that at higher pressures, 10- and 5-µm particles were more productive. A later study used Monte Carlo simulations of 1000 randomly selected separation systems to draw more general conclusions [16]. It was found that it is almost always beneficial to use shorter columns with higher pressure drops. Moreover, the dependence of productivity on packing particle size, as mentioned above, was verified and can be summarized as follows: (1) if the pump’s maximum flow rate is the limiting factor, use smaller particle-size packing; but (2) if the system pressure is the limiting factor, use larger-particle-size (≤ 40 µm) packing [16–18].

The model compounds in this study are the proton-pump inhibitor omeprazole and the antiepileptic levetiracetam (an enantiomer of etiracetam). Racemic omeprazole and its biologically more potent *S*-enantiomer (esomeprazole) are synthesized without using chromatography [19]. Omeprazole was selected, because it has previously been studied from the perspective of chromatographic separation and optimization [3, 8, 17]. Levetiracetam was selected, because it is currently produced using large-scale continuous chromatography [6] and it was the first active pharmaceutical ingredient (API) produced this way [20]. An integrated synthesis and chromatographic process for resolving levetiracetam from etiracetam was designed by UCB in the 1990s and 2000s [6], and chromatography was found to be the most economic method. Smaller particles were deemed uneconomic due to the higher cost of the material and higher required pressures in the chromatographic system [3].

The aim of this investigation is to determine how the Dynamic Axial Compression (DAC) column should be packed (i.e., determine the appropriate column length and stationary-phase particle size) to achieve optimal productivity. For over 20 years, preparative batch LC has been dominated by the DAC mode, in which a hydraulically actuated piston allows any column length to be selected, while the column diameter is fixed [21]. The process is first optimized using numerical modeling, but, since numerical optimization is tedious from a practical perspective, a simpler statistical optimization approach, i.e., the Taguchi method, is evaluated as an alternative. The Taguchi method exploits special standard orthogonal arrays, which has been successfully applied in many manufacturing industries and experimental designs [22], and it is well suited for discrete variables. Experimentally, the two separation processes were performed at maximum allowed backpressures of 80 and 200 bar to determine whether pressure affected the optimal column length and stationary-phase particle size in these cases.

## Theory

### Column Model

This study used the equilibrium–dispersive model equivalent to general rate (EDEG) model, which is based on the equilibrium–dispersive (ED) model [5, 9], described as follows:

1 $$ {\frac{{\partial c_{i} }}{\partial t} + \frac{{(1 - \varepsilon_{t} )}}{{\varepsilon_{t} }}\frac{{\partial q_{i} }}{\partial t} + \frac{u}{{\varepsilon_{t} }}\frac{{\partial c_{i} }}{\partial z} = \frac{\partial }{\partial z}\left( {D_{a,z} \frac{{\partial c_{i} }}{\partial z}} \right)}, $$

where *c*_*i*_ and *q*_*i*_ are the concentrations of the mobile and stationary phases, respectively, *u* is the superficial velocity, *ε*_t_ is the total external porosity, *t* is time, and *D*_*a,z*_ is an apparent dispersion coefficient whose value can be determined from the measured height equivalent to the theoretical plate (HETP). For preparative chromatography, the column works in the nonlinear part of the isotherm curve and *D*_*a,z*_ depends indirectly on the sample concentration [21]. When the transport-dispersive model is to be used, one must correctly calculate the effective mass transfer coefficient, which also depends on concentration [23]. In the present work, we analyzed a preparative process for species separation and decided to apply a version of the EDEG model proposed by Antos et al. [21] and Kaczmarski et al. [23, 24].

In solving Eq. (1), the apparent dispersion coefficient is calculated from the following relationship, for the first and second components:

2 $$ {D_{a,z} = \frac{{D_{\text{L}} \varepsilon_{\text{e}} }}{{\varepsilon_{\text{t}} }} + \left( {\frac{{k_{1} }}{{1 + k_{1} }}} \right)^{2} \frac{{u^{2} d_{\text{p}} }}{{\varepsilon_{\text{t}} \varepsilon_{\text{e}} F_{\text{e}} 6}}\left[ {\frac{{d_{\text{p}} }}{{10D_{\text{eff}} }} + \frac{1}{{k_{\text{ext}} }}} \right]}, $$

where *ε*_e_ is external porosity, *D*_L_ is an axial dispersion coefficient, and *k*_1_, *F*_e_, and *D*_eff_ can be expressed as:

3 $$ {k_{1} = F_{\text{e}} \left( {\varepsilon_{\text{p}} + \left( {1 - \varepsilon_{\text{p}} } \right)\frac{\partial q}{\partial c}} \right)},\quad {F_{\text{e}} = \frac{{1 - \varepsilon_{\text{e}} }}{{\varepsilon_{\text{e}} }}},\quad {\text{and}}\quad {D_{\text{eff}} = \frac{{\varepsilon_{\text{p}} D_{\text{m}} }}{\tau },} $$

respectively, where *D*_m_ is diffusivity, *k*_ext_ is a mass transfer coefficient, *d*_p_ is particle diameter, *τ* is tortuosity, *ε*_p_ is pore porosity, and ∂*q*/∂*c* is approximated using the slope at *C *= 0. When calculating *D*_eff_, it is assumed that the surface diffusion can be ignored. The external porosity, *ε*_e_, is assumed to be 0.4. The tortuosity coefficient was obtained from the following relationship:

4 $$ {\tau = \frac{{(2 - \varepsilon_{\text{p}} )^{2} }}{{\varepsilon_{\text{p}} }}}. $$

In this study, a bi-Langmuir-like adsorption–desorption kinetic model was used for the enantiomeric separation of omeprazole; it can be described as:

5 $$ \frac{{\partial q_{i}^{j} }}{\partial t} = k_{{{\text{a}},i}}^{j} C_{i} \left( {q_{{{\text{s}},i}}^{j} - q_{1}^{j} - q_{2}^{j} } \right) - k_{{{\text{d}},i}}^{j} q_{i}^{j} , $$

where *k*_a_ and *k*_d_ are the rate constants for adsorption and desorption, respectively, *q*_s_ is the monolayer saturation capacity, and $q_{i}^{j}$ is the amount of component *i* adsorbed on site *j*. The total amount of adsorbed compound is:

6 $$ q_{i} = q_{i}^{1} + q_{i}^{2} , $$

where *q*_*i*_ is the adsorbed amount of component *i* and the superscript indicates the first site or second site.

For the enantiomeric separation of etiracetam, the following competitive bi-Langmuir isotherm model was applied:

7 $$ q_{i} = \frac{{q_{\text{ns}} K_{\text{ns}} C_{i} }}{{1 + K_{\text{ns}} \left( {C_{1} + C_{2} } \right)}} + \frac{{q_{\text{es}} K_{\text{es}} C_{i} }}{{1 + K_{{{\text{es}},1}} C_{1} + K_{{{\text{es}},2}} C_{2} }}, $$

where *q*_ns_ and *q*_es_ are the saturation capacities and *K*_ns_ and *K*_es_ are the association equilibrium constants of the nonselective and selective adsorption sites, respectively. This isotherm model assumes two types of adsorption sites: the first type (first term) behaves identically toward the two enantiomers, while the second type (second term) is enantioselective and responsible for chiral separation. The model has been widely and successfully applied to describe adsorption isotherms of chiral solutes; for example, the 1-indanol enantiomers [25]. The benefits of using bi-Langmuir adsorption model are that it has a foundation in the physiochemical adsorption process, but a drawback is that it contains more parameters, thus making the numerical determination more complex.

The discussed model was solved with typical initial and boundary conditions and the kinetic parameters were calculated as follows. The mass transfer coefficient, *k*_ext_, was evaluated from the Wilson–Geankoplis correlation [26]:

8 $$ Sh = \frac{1.09}{{\varepsilon_{\text{e}} }}Re^{1/3} Sc^{1/3} , $$

where *Sh* is the Sherwood number, *Re* the Reynolds number, and *Sc* the Schmidt number:

9 $$ {\text{Sh}} = \frac{{k_{\text{ext}} d_{\text{p}} }}{{D_{\text{m}} }}, \, \text{Re} = \frac{{u\rho d_{\text{p}} }}{\eta }{\text{ and Sc}} = \frac{\eta }{{\rho D_{\text{m}} }}. $$

The diffusivity, *D*_m_ (m^2^ s^−1^), was calculated from the Wilke–Chang model [27]:

10 $$ {D_{\text{m}} = 1.17 \times 10^{ - 16} \;T\frac{{(\alpha \cdot M_{\text{w}} )^{0.5} }}{{\eta \cdot V_{i}^{0.6} }},} $$

where *T* is temperature (K), *M*_w_ is the solute molar mass (kg mol^−1^), *η* is viscosity (Pa × s), *α* = 1.9 for methanol and 1 for 60/40 ethanol/heptane, *V*_*i*_ is the molar volume in the mobile phase (m^3^ mol^−1^), and *V*_*i*_ = 368.5 × 10^−3^ (m^3^ kmol^−1^) for omeprazole and 194.5 × 10^−3^ (m^3^ mol^−1^) for etiracetam. Finally, the axial dispersion coefficient is obtained from the following relationship:

11 $$ {D_{\text{L}} = 0.7D_{\text{m}} + 0.5ud_{\text{p}} }. $$

In this study, the adsorption isotherm was estimated using the inverse method [28, 29], solved using orthogonal collocation on finite elements [24]. The chosen base experimental concentration profiles were estimated by minimizing the sum of squared differences between the experimental and calculated elution profiles.

### Optimization

As objective function in the optimization, the productivity (Pr) (defined as gram product collected per minute), was used. In this study, the optimization problem comes down to the following expression:

12 $$ {\text{Max}}(\text{Pr}_{i} ) = f(u,t_{\text{inj}} ,d_{\text{p}} ,L). $$

As the optimization problem expressed by Eq. (12) was quite complex, the hybrid method of simulated annealing coupled with a simplex algorithm was applied [30]. This hybrid optimization tool, which combines the stochastic method of simulated annealing with a deterministic simplex algorithm, is suitable for solving difficult optimization problems in which the desired global optimum is hidden among many local optima. The detailed algorithm and a study of the effectiveness of the applied optimization method were presented by Kaczmarski and Antos [31]. However, this numerical optimization of the chromatographic separation is time-consuming; therefore, a faster algorithm based on design of experiments (DoE) was also investigated.

The DoE method allows one to conduct just a few experiments to describe the system variance. In DoE, all factors (in this case, superficial fluid velocity, injection time, column length, and particle diameter) are studied at different levels (i.e., low, medium, and high). As the total number of required experiments increases rapidly with the number of factors, reduced design schemas are often employed. The Taguchi model is a special orthogonal array approach that drastically reduces the number of needed experiments [32]. In this study, four factors were considered at three levels. For such cases, Taguchi proposes the *L*_9_ orthogonal array, which reduces a full-factor design of 81 (3^4^) experiments to only nine (see Tables S1–S4 in Electronic Supplementary Material).

The Taguchi optimum can be calculated as:

13 $$ (\text{Pr} /L)_{\text{opt}} = \overline{T} + \left( {\overline{A}_{\text{max}} - \overline{T} } \right) + \left( {\overline{B}_{\text{max}} - \overline{T} } \right) + \left( {\overline{C}_{\text{max}} - \overline{T} } \right) + \left( {\overline{D}_{\text{max}} - \overline{T} } \right), $$

where $\overline{A}_{\text{max}}$, $\overline{B}_{\text{max}}$, $\overline{C}_{\text{max}}$ and $\overline{D}_{\text{max}}$ are the highest average effects of the factors corresponding to the optimal values of the factors; and $\overline{T}$ is the grand average of productivity, obtained by averaging the results of all trial combinations of factors and levels:

14 $$ \overline{T} = \frac{1}{9}\sum\limits_{i = 1}^{9} {(\text{Pr}_{i} /L)} , $$

where Pr_*i*_ is the productivity of individual trials. The average effect of a factor at a given level is calculated by averaging all productivities containing the factor level of interest:

15 $$ \begin{aligned} \overline{{A_{i} }} = \frac{1}{3}\sum\limits_{j = 1}^{3} {(\text{Pr}_{i,j} } /L),\quad \overline{{B_{i} }} = \frac{1}{3}\sum\limits_{j = 1}^{3} {(\text{Pr}_{i,j} /L)} , \hfill \\ \overline{{C_{i} }} = \frac{1}{3}\sum\limits_{j = 1}^{3} {(\text{Pr}_{i,j} /L)} \quad {\text{and}}\quad \overline{{D_{i} }} = \frac{1}{3}\sum\limits_{j = 1}^{3} {(\text{Pr}_{i,j} } /L), \hfill \\ \end{aligned} $$

where $\overline{A}_{i}$, $\overline{B}_{i}$, $\overline{C}_{i}$ and $\overline{D}_{i}$ are the average effects (i.e., productivity) of the considered factors at the *i*th level; and Pr_*i,j*_ is the productivity calculated for the considered factor at the *i*th level in accordance with the *L*_9_ array (see Tables S1–S4).

## Experimental

### Apparatus and Chemicals

The solutes used in this study were *R*/*S*-omeprazole and *R*/*S*-etiracetam obtained from AstraZeneca (Mölndal, Sweden) and UCB Pharma (Bruxelles, Belgium), respectively. As eluent, HPLC-grade (99.99%) methanol from Fisher Scientific (Loughborough, UK) was used for omeprazole [17] and 60/40% (v/v) ethanol/heptane was used for etiracetam [33]. The ethanol was analytical grade (99.90%) from VWR International (Fontenay-sous-Bois, France) and the heptane was HPLC grade (99%) from Fisher Scientific (Loughborough, UK). In all experiments, 1,3,5-tri-tert-butylbenzene (97%) from Sigma-Aldrich (Steinheim, Germany) was used to estimate the void volume. The analytical scale experiments were performed on Agilent 1100 and 1200 HPLC systems (Palo Alto, CA, USA) consisting of a binary pump, a preparative auto-sampler (900 µL, max. 200 bar) and a diode array UV detector. At analytical scale, 0.46-cm-i.d. Kromasil AmyCoat columns from Akzo Nobel Pulp and Performance Chemicals AB (Bohus, Sweden), 10-, 15-, and 25-cm long and packed with 10- and 25-µm particles, were used. An additional 10 × 0.46-cm AmyCoat column packed with 5-µm particles was used in the validation experiments. The column temperature was kept at 23.0 °C by immersing the columns in a temperature-controlled water bath. The pilot-scale experiments were performed using a Packer LC50.340 VE100 PS TH column (Novasep, Boothwyn, PA, USA), 50-mm i.d., packed to a bed height of 105 mm with AmyCoat 5-µm particles (same batch as used in the analytical columns), together with two K-1800 preparative pumps and a K-2600 UV detector (Knauer, Berlin, Germany).

### Procedures

The holdup volumes were estimated on all columns for both eluents by injecting 5 µL of diluted 1,3,5-tri-tert-butylbenzene three times at a flow rate of 2 mL min^−1^ (except at pilot scale, at which the flow rate was 205 mL min^−1^) when detecting at 220 nm. The average elution volume was considered to be the void volume of the column. Analytical injections of 5 µL of 1 g L^−1^ omeprazole dissolved in MeOH and of 0.35 g L^−1^ etiracetam dissolved in 60/40% (v/v) ethanol/heptane were performed and recorded at 220 nm. The pressure/flow rate dependence was determined at 1–5 mL min^−1^ for all column lengths and packing material particle sizes using pure methanol as well as 60/40% (v/v) ethanol/heptane as the eluent. The system pressure contributions of the Agilent 1100 and 1200 systems were also measured without columns at 1–5 mL min^−1^ using both eluents. Overloaded duplicate samples of 50, 100, 200, 400, 600, and 900 µL of 30 g L^−1^ omeprazole and triplicate samples of 50, 75, 100, 150, 200, 250, 300, and 350 µL of 60 g L^−1^ etiracetam were injected into each column. A flow rate of 2 mL min^−1^ was maintained for both substances. Chromatograms were recorded at 345 nm for omeprazole and 260 nm for etiracetam.

### Calculations

The adsorption parameters needed to solve Eq. (1) were estimated from the experimental overloaded elution profiles using the inverse method. Before calculating the adsorption parameters using the inverse method, the UV absorbance was converted to concentration. This was done by fitting the UV response to the elution profile and requiring that the mass balance equation be fulfilled [34].

The optimum productivity was calculated as a function of the flow rate, column length, packing material diameter, and injection time. As constraints, the maximum allowed backpressure was set to 80 or 200 bar, and the purity of the products to 99%.

In the calculations, the particle size was varied between 5 and 25 µm, the flow rate was set to 5 mL min^−1^, the pressure constraint was as calculated from the experimental measurements, the column length was varied between 100 and 250 mm, and the injection volume was limited to a maximum of 900 µL. All calculations were conducted assuming the column to have an inner diameter of 4.6 mm. The particle diameter, superficial fluid velocity, and column length were limited by the pressure drop, which is the sum of the column pressure drop and the pressure drop contributed by the system. The pressure drop of the Agilent system was approximated by a polynomial relationship based on experimental data and the column pressure drop was calculated using the Blake–Kozeny equation.

In the numerical optimization, the particle diameter and column length were considered continuous variables in the optimization, so they were rounded to the nearest real value after optimization.

In the Taguchi approach, four variables (factors) were considered at three levels, as follows: superficial velocity of the mobile phase (*u*_max_, 2/3 *u*_max_, and 1/3 *u*_max_), injection volume (0.9, 0.6, and 0.3 cm^3^), column length (25, 15, and 10 cm), and particle diameter (25, 10, and 5 µm). The *u*_max_ is dependent on the eluent and the maximum allowed pressure drop (80 or 200 bar) in the separation.

## Results and Discussion

### Numerical Model Validation

Previously, the equilibrium–dispersive (ED) column model has been used to describe the enantiomeric separation of omeprazole [17]. However, the analytical peaks for omeprazole were tailing. To deduce whether this peak tailing is due to thermodynamic (overloading) reasons, the column load of omeprazole was reduced from 25 µg to 1.25 µg on a 250 × 4.6 mm column without any reduction in peak tailing, see Fig. S1 in the Electronic Supplementary Material. We also noted that increasing the flow rate from 0.25 to 2 mL min^−1^ resulted in increased tailing, see Fig. S2 in the Electronic Supplementary Material. We, therefore, conclude that the peak tailing occurs due to kinetic reasons, namely, a slow adsorption–desorption process. From a model perspective, the kinetic tailing is handled using a kinetic representation of the adsorption isotherm, see Eq. (5).

**Fig. 1:**
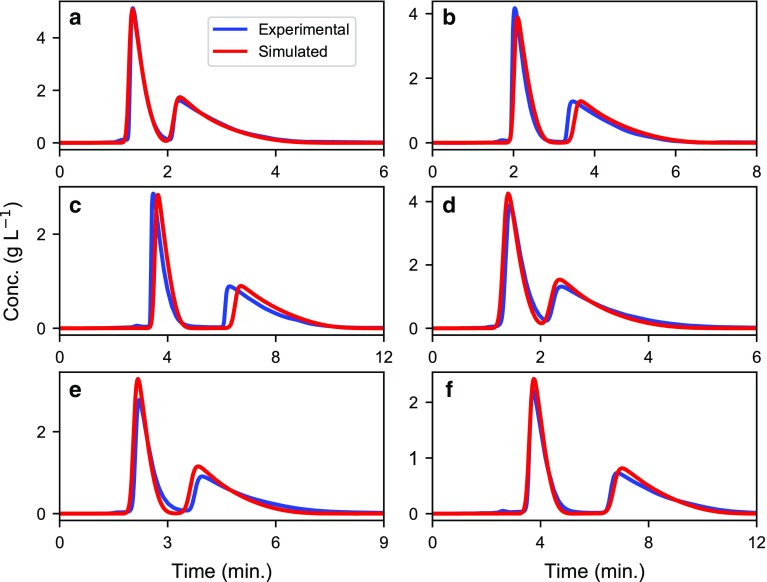
Experimental chromatograms (blue lines) and model predictions (red lines) of the enantiomeric separation of omeprazole. Flow rate, 2 mL min^−1^; injection volume, 200 μL. Columns: **a** 10 μm, 4.6 × 100 mm; **b** 10 μm, 4.6 × 150 mm; **c** 10 μm, 4.6 × 250 mm; **d** 25 μm, 4.6 × 100 mm; **e** 25 μm, 4.6 × 150 mm; and **f** 25 μm, 4.6 × 250 mm

**Fig. 2:**
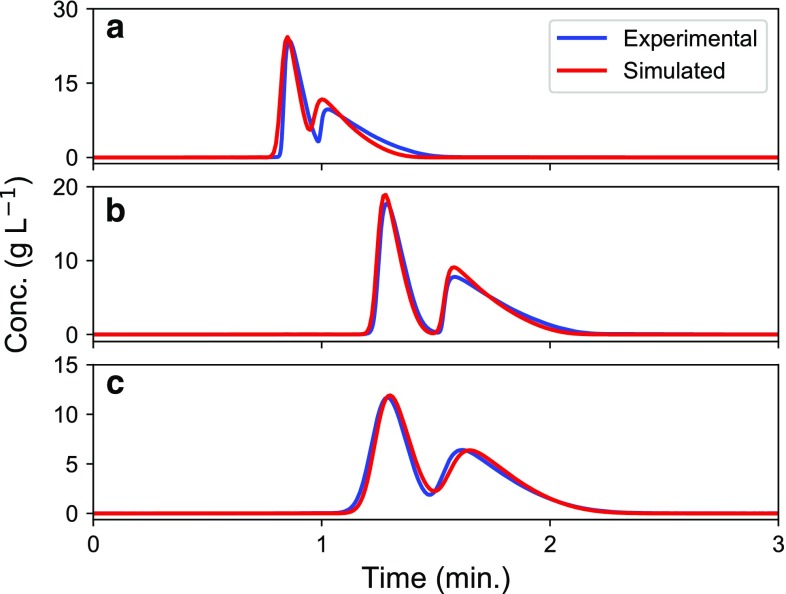
Experimental chromatograms (blue lines) and model predictions (red lines) of the enantiomeric separation of etiracetam. Flow rate, 2 mL min^−1^; injection volume, 150 μL. Columns: **a** 5 μm, 4.6 × 100 mm; **b** 10 μm, 4.6 × 150 mm; and **c** 25 μm, 4.6 × 150 mm

To calculate the elution profiles more accurately, the EDEG model was used (see subsection “Column Model” in the “Theory” section). The inverse method was used to estimate the adsorption isotherm parameters for the enantiomeric separation of omeprazole and etiracetam at different column lengths and particle sizes. Figure 1 presents the experimental chromatograms and the model predictions for the enantiomeric separation of omeprazole, while Fig. 2 presents the corresponding experimental and predicted results for etiracetam. Inspecting Figs. 1 and 2, we can conclude that the numerical models describe the experimental data well.

**Fig. 3:**
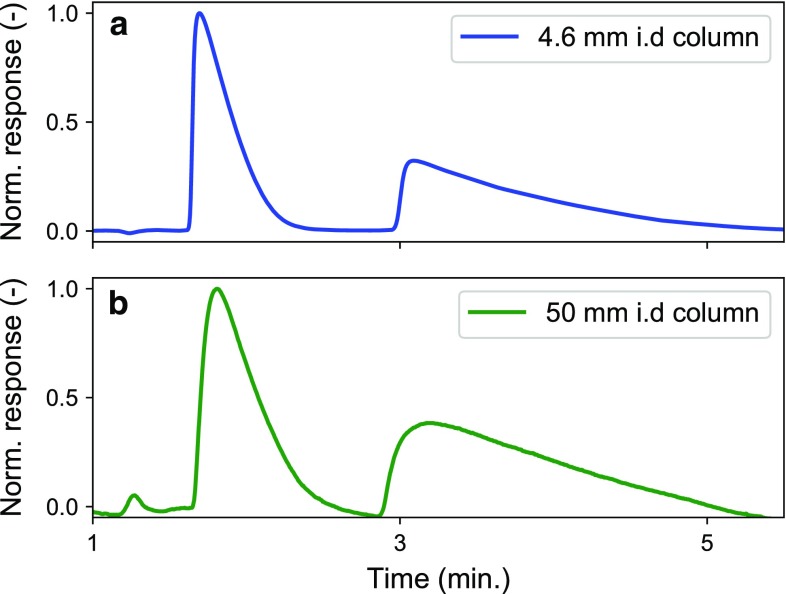
Enantiomeric separation of omeprazole on AmyCoat columns at analytical scale (top, blue line) and pilot scale (bottom, green line). Analytical separation on a 100 × 4.6 mm column using a 85-µL sample of 30 g L^−1^ racemic omeprazole injected at a flow rate of 1.7 mL min^−1^; pilot separation on a 105 × 50 mm column using a 10-mL sample of 30 g L^−1^ racemic omeprazole injected at a flow rate of 205 mL min^−1^

To investigate potential scale-up issues, the enantiomeric separation of omeprazole conducted on a 4.6-mm-i.d. column was compared with separation experiments conducted on a 50-mm-i.d. pilot-scale column, corresponding to a 118-times scale-up (see Fig. 3). Both columns, approximately 100-mm long, were packed with 5-µm AmyCoat packing. In Fig. 3a, the separation on the 4.6-mm-i.d. column is plotted; this separation was performed with an 85-µL injection of 30 g L^−1^ omeprazole at a flow rate of 1.7 mL min^−1^. Figure 3b shows the corresponding pilot-scale separation, performed with a 10-mL injection of 30 g L^−1^ omeprazole at a flow rate of 205 mL min^−1^. As it can be seen, the experiments conducted at analytical scale and pilot scale differed only slightly in their results, so it can be concluded that this process is likely scalable and that our model could be used to predict elution profiles.

### Optimal Column Length and Particle Size

**Fig. 4:**
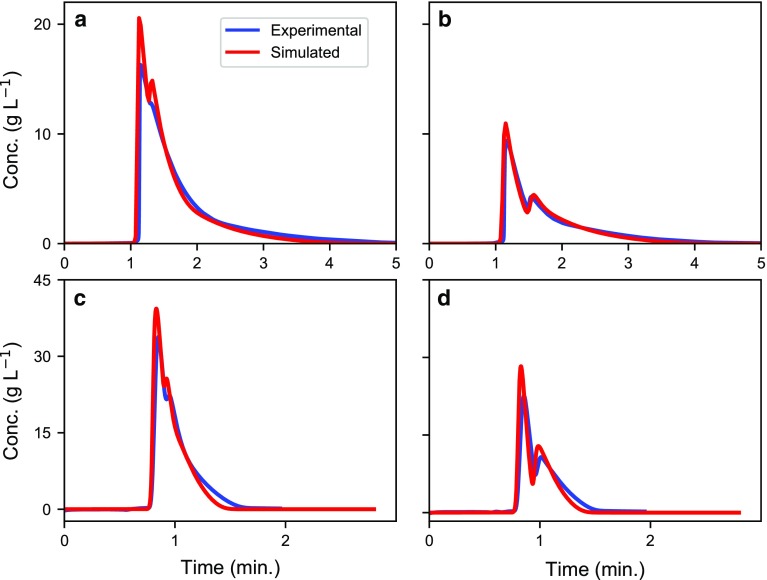
Experimental (blue lines) and calculated (red lines) optimal conditions for the enantiomeric separation of omeprazole and etiracetam at 80 bar for the first- and second-eluted enantiomers. Optimal conditions for: **a** first-eluted enantiomer of omeprazole, **b** second-eluted enantiomer of omeprazole, **c** first-eluted enantiomer of etiracetam, and **d** second-eluted enantiomer of etiracetam

**Fig. 5:**
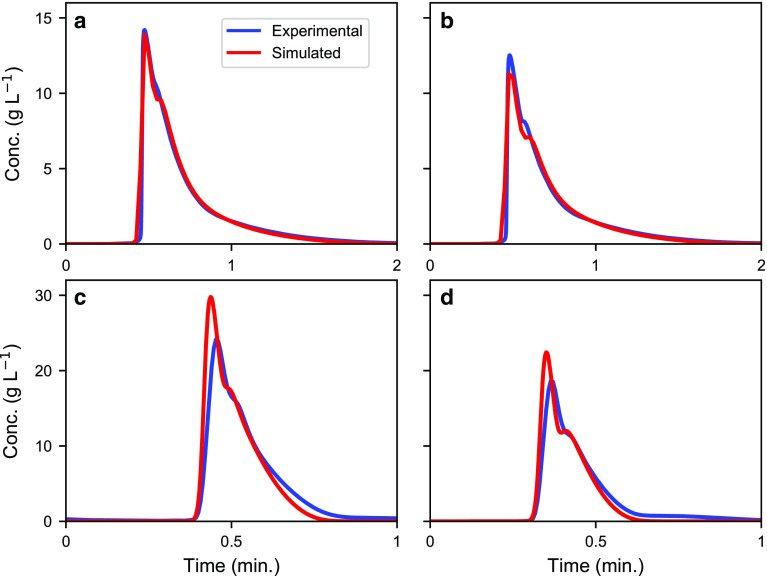
Experimental (blue lines) and calculated (red lines) optimal conditions for the enantiomeric separation of omeprazole and etiracetam at 200 bar for the first- and second-eluted enantiomers. Optimal conditions for: **a** first-eluted enantiomer of omeprazole, **b** second-eluted enantiomer of omeprazole, **c** first-eluted enantiomer of etiracetam, and **d** second-eluted enantiomer of etiracetam

Based on the model validation results, the enantiomeric separation of omeprazole and etiracetam was numerically optimized and experimentally confirmed. Table 1 presents the optimal experimental settings as well as the calculated productivity. Figures 4, 5 present the calculated optimal conditions for the enantiomeric separation of omeprazole and etiracetam at 80 and 200 bars, respectively, for the *R* and *S* enantiomers with overlaid experimental verification. In both cases, relatively good agreement was obtained.

**Table 1:** Optimum conditions for first- and second-eluted enantiomers of omeprazole and etiracetam, respectively

Compound	Target	Δ*P*_max_ (bar)	Method	*L* (cm)	*d*_p_ (µm)	*u* (cm min^−1^)	*t*_inj_ (min)	Pr × 10^2 a^ (g min^−1^)	Pr × 10^2 b^ (g min^−1^)	SP × 10^3^ (g cm^−1^)
Omeprazole	S (1)	80	T	10	25	8.32	0.651	0.8336	0.9376	0.7642
			SS	10	5	12.20	0.342	1.337	–	0.8363
		200	T	10	5	30.08	0.180	1.678	1.616	0.4260
			SS	10	5	29.55	0.119	1.995	–	0.5150
	R (2)	80	T	10	5	8.32	0.651	0.5940	0.5762	0.5445
			SS	10	5	12.40	0.186	0.8417	–	0.5177
		200	T	10	5	30.08	0.180	1.296	0.9800	0.3291
			SS	10	5	29.59	0.1027	1.345	–	0.3469
Etiracetam	S (2)	80	T	15	5	4.80	1.128	2.535	2.526	3.792
			SS	10	10	12.32	0.087	4.659	–	2.695
		200	T	10	5	25.0	0.217	4.178	3.793	1.199
			SS	10	10	29.47	0.040	6.542	–	1.582
	R (1)	80	T	15	5	4.80	0.376	3.392	2.576	5.073
			SS	10	10	12.35	0.140	6.270	–	3.618
		200	T	15	5	16.7	0.324	6.868	8.931	1.969
			SS	10	10	23.60	0.062	7.463	–	2.254

Two optimization methods were used: *T* Taguchi and *SS* simulated annealing + simplex. The numbers 1 and 2 under “Target” indicate the first- and second-eluting enantiomers

^a^In the Taguchi method, the productivity is calculated using the column model with optimum run conditions predicted using the Taguchi method

^b^Productivity is estimated using the Taguchi method, Eq. (13)

The optimum particle size for omeprazole at 80 and 200 bar is 5 µm, and the maximum productivity was found at the minimum allowed column length of 10 cm. When the maximum allowed pressure was increased from 80 to 200 bar, this resulted in an approximately 1.5 times higher productivity, while the solvent consumption decreased 0.6 times. The reduced solvent consumption with increased pressure is in line with the previous findings [17].

For etiracetam, the optimum packing at 80 and 200 bar is 10 µm and, as in the case of omeprazole, the shortest column of 10 cm should be used at both pressures. Increasing the maximum allowed pressure from 80 to 200 bar led to approximately 1.4 times higher productivity, while the solvent consumption decreased by 0.6 times. This clearly indicates that pressure is the most important factor in increasing the productivity, because it allows the operational flow rate to increase. This also suggests that using smaller particles will result in more productive processes.

From Table 1, we also see, as suspected, that the productivity is always higher for the first-eluted compound (i.e., *S*-omeprazole and *R*-etiracetam), for both systems. However, the etiracetam process is approximately 4–5 times more productive than the omeprazole process, mainly because the cycle time is much shorter for etiracetam than for omeprazole. Other factors that increase the productivity for etiracetam are that the separation system is more efficient and that the sample concentration of etiracetam is double that of omeprazole due to solubility reasons.

### Taguchi vs. Classical Numerical Optimization

The Taguchi approach was investigated to see whether it could speed up the optimization process. From a practical perspective, the Taguchi method could be interesting as an alternative/complement to touching-band optimization, because it could simplify the selection of column length, packing material, packing material particle size, etc.

**Fig. 6:**
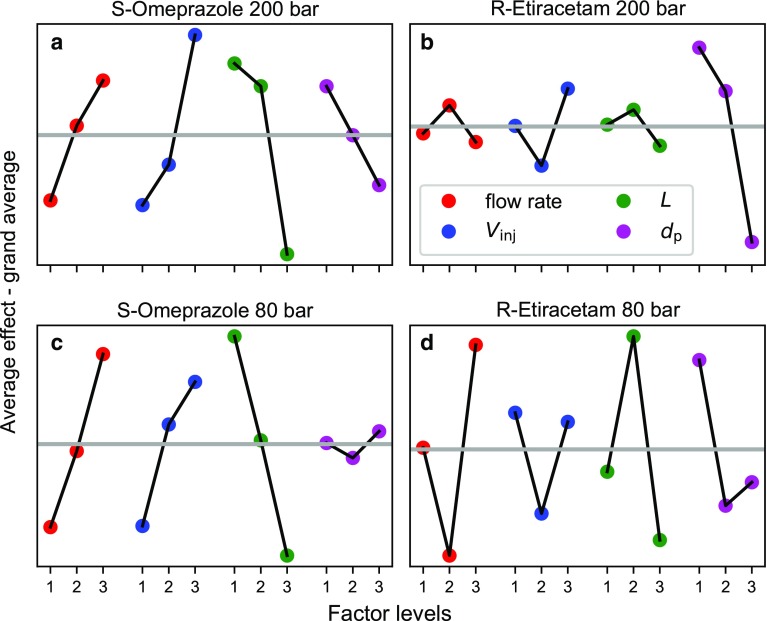
Plot of factor average effect minus the grand average in Taguchi optimization, optimized system: **a** omeprazole (*S*), pressure restriction 200 bar; **b** etiracetam (*R*), pressure restriction 200 bar; **c** omeprazole (*S*), pressure restriction 80 bar; **d** etiracetam (*R*), pressure restriction 80 bar. The gray line is the grand average

Here, the Taguchi method required only nine experiments to optimize the column length, stationary-phase packing material particle size, injection volume, as well as flow rate (see Tables S1–S4 in Electronic Supplementary Material for more information). The optimal decision variables and productivity estimated using Taguchi optimization are presented in Table 1. In Fig. 6, the normalized average effects of all decision variables are plotted for the optimization of *S*-omeprazole and *R*-etiracetam at 200 and 80 bar.

In the omeprazole case, roughly the same optimal conditions were predicted using both the numerical and Taguchi methods. At 80 bar, the Taguchi method suggested using smaller packing than did the numerical method. However, the average effects of stationary-phase packing particle diameter calculated using the Taguchi method were only slightly worse for 5-µm than for 25-µm particles (see Fig. 6c). Inspecting the productivity, in Table 1, the classical numerical method predicted much higher productivity for the 80 bar omeprazole separation for both enantiomers than did the Taguchi method. This is because Taguchi optimization was flow limited, i.e., operating at the maximum allowed flow rate. The maximum allowed flow rate in the Taguchi method was set to the maximum allowed flow rate for the longest column (25 cm) packed with the smallest stationary-phase packing particles (5 µm). When the Taguchi method was flow limited, the maximum allowed flow rate could not be increased by decreasing the column length or increasing the packing material particle size, as can be done in classical numerical optimization. Therefore, the velocities obtained from Taguchi and from classical numerical optimization differed from each other.

In the etiracetam case, larger differences in optimal separation systems were found between the classical numerical and Taguchi methods than in the omeprazole case (Table 1). In this case, the Taguchi method suggested that longer columns packed with smaller packing particles should be used than did the numerical method. To explain these differences, first, we note that the separation was flow limited (except for *R*-etiracetam at 200 bar) in the Taguchi optimization. As a consequence, longer columns and smaller packing were found as optimum by the Taguchi method (see Fig. 6b, d). In the 200 bar case, the column length had very small impact on the optimum; see Fig. 6b.

The Taguchi approach is very useful and successful, mainly in areas where optimal values of discrete decision variables, such as packing particle diameter and column length, are to be determined. The Taguchi method is easy to apply in such cases, but it may be difficult to establish appropriate Taguchi design spaces in more complex optimizations such as those presented here. To conclude, the Taguchi approach could be recommended for relatively simple optimization or pre-optimization to help in selecting column packing, etc. For accurate and complicated optimizations, however, classical numerical methods are still preferred.

## Conclusion

In this study, we have considered, given a column of a certain diameter, what column length should be selected and what sized particles that the column should be packed with to achieve maximum productivity. We also compared advanced numerical optimization based on a mechanistic model with empirical optimization, i.e., the Taguchi method, with the latter being more readily available in the laboratory to the general chromatographer.

Both separation systems were numerically optimized to derive the optimal packing material particle size, column length, and flow rate. Maximum allowed backpressures of 80 and 200 bar were investigated. Preparative chromatography is often conducted using large-particle-diameter packing material and a column length of 25 cm. Here, we demonstrated that, in the studied batch chromatography cases, shorter columns were more suitable when using packing materials with smaller stationary-phase particle sizes. In both investigated cases, a column length of 10 cm was found to be optimal. We also demonstrated that increasing the maximum allowed backpressure 2.5 times resulted in approximately 1.5 times greater productivity and a 0.6 times reduction in solvent consumption.

In this study, we also used the Taguchi method, a chemometric method that can work strictly from experimental data. The Taguchi method is easy to learn and use for the practical chromatographer. It is very suitable for quickly determining optimal discrete parameters or pre-estimating an objective function, as exemplified in this study by determining what column length and particle diameter should be used to achieve the highest productivity. In this study, we noted that short columns packed with smaller particles were preferable to longer columns packed with larger particles. The optimum process conditions were not exactly found, this is why we still recommend classical numerical optimization for the most accurate process optimization. However, this study clearly demonstrates that quite acceptable predictions can be achieved with less numerical effort.

## Electronic supplementary material

Below is the link to the electronic supplementary material.

Supplementary material 1 (DOC 638 kb)
