# Supplementary material for: Optimizing Column Length and Particle Size in Preparative Batch Chromatography Using Enantiomeric Separations of Omeprazole and Etiracetam as Models: Feasibility of Taguchi Empirical Optimization
Source: Chromatographia. 2018 Apr 25;81(6):851–60. doi: 10.1007/s10337-018-3519-z (PMC5972160; doi:10.1007/s10337-018-3519-z)
Supplement: Supplementary file 1 — Supplementary material 1 (DOC 638 kb) [file 10337_2018_3519_MOESM1_ESM.doc]

**Optimizing Column Length and Particle Size in Preparative Batch Chromatography using Enantiomeric Separations of Omeprazole and Etiracetam as Models: Feasibility of Taguchi Empirical Optimization**

Jörgen Samuelsson*^1^, Marek Leśko^2^, Martin Enmark^1^, Joakim Högblom^3^, Anders Karlsson^4^, Krzysztof Kaczmarski*^2^

^1^ Department of Engineering and Chemical Sciences, Karlstad University, SE-651 88 Karlstad, Sweden

^2^ Department of Chemical Engineering, Rzeszow University of Technology, PL-35 959 Rzeszów, Poland

^3^ Akzo Nobel Pulp and Performance Chemicals AB, SE-445 80 Bohus, Sweden

^4^ AstraZeneca R&D, SE-431 83 Mölndal, Sweden

^*^Corresponding authors

K. Kaczmarski: tel.: +48 17 865 1295; fax: +48 17 854 3655; E-mail: kkaczmarski@prz.edu.pl

J Samuelsson: tel.: +46 54 700 1620; fax: +46 54 700 2040; E-mail: jorgen.samuelsson@kau.se

# Electronic Supplementary Material

This section presents the kinetic investigation and the Taguchi optimization. The kinetic investigation is presented in Fig. S1 (different loads) and Fig. S2 (different flow rates). In Table S1 the Taguchi optimization schemes for omeprazole, for the 200-bar and Table S2 for the 80-bar pressure restriction is presented. Corresponding optimization schemes are presented for etiracetam in Table S3 for the 200-bar and Table S4 for the 80-bar pressure restriction.

## Kinetic investigation

To investigate the origin of the peak tailing two different experiments were conducted. First different load were investigated, see Fig. S1. Secondly different flow rate were investigated, see Fig. S2.


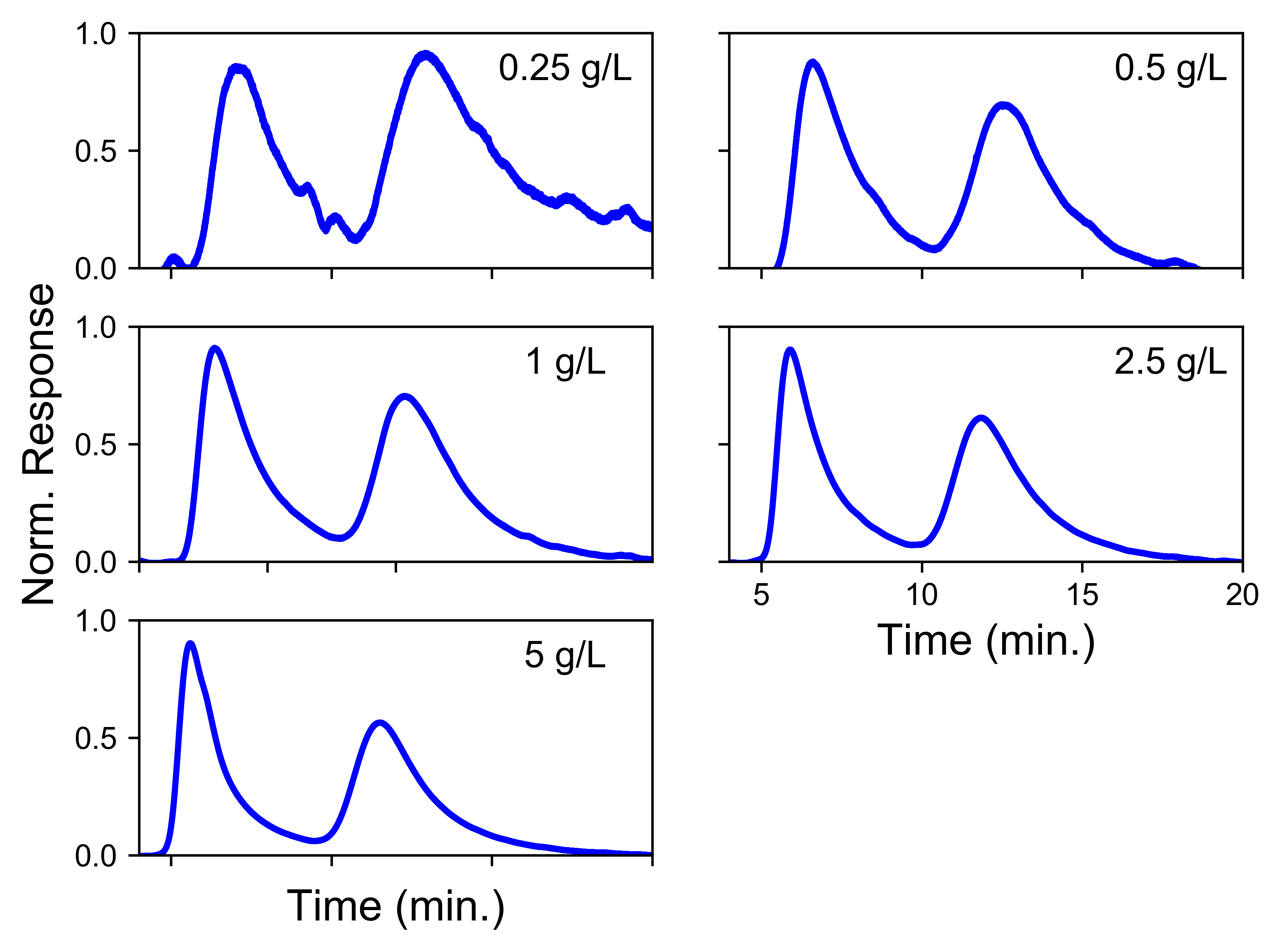


**Figure S1**: Kinetic load experiments AmyCoat 25 µm (250 x 4.6 mm) column. 5 µL of 0.25, 0.5, 1.0, 2.5 and 5 g L^-1^ omeprazole were injected (see text in figures); flow rate of 2 mL min^-1^.


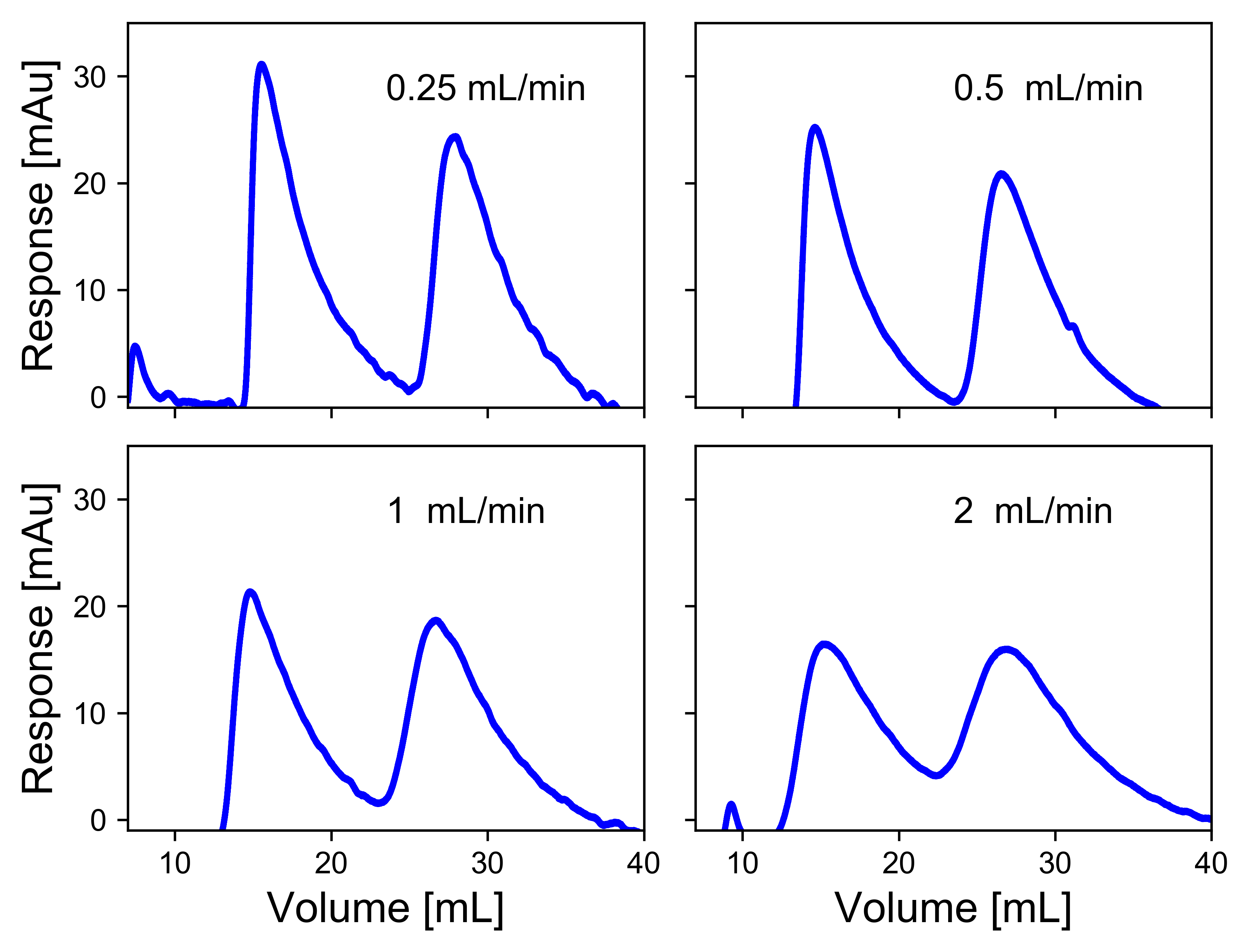


**Figure S2**: Kinetic flow rate experiments on the AmyCoat 25 µm (250 x 4.6 mm) column. In all experiments 5 µL of 1.0 gL-1 omeprazole were injected, at different flowrates: 0.25, 0.5, 1.0 and 2.0 mL min^-1^ (see text in figures).

## Taguchi optimization

**Table S1** L_9_(3^4^) Taguchi design space used for optimizing first- and second-eluted compounds of omeprazole; the design space is intended for the 200-bar pressure restriction.

| **Trial** | **A**  **u [cm min^–1^]** | **B**  **V_inj_ → t_inj_ [cm^3^]** | **C**  **L [cm]** | **D**  **d_p_ [µm]** | **Results: Pr/L × 10^4^ [g (min × cm)^–1^]** | |
| --- | --- | --- | --- | --- | --- | --- |
|  |  |  |  |  | **Compound 1 (S)** | **Compound 2 (R)** |
| **1** | 1 (10.03) | 1 (0.3) | 1 (10) | 1 (5) | 8.391 | 7.447 |
| **2** | 1 | 2 (0.6) | 2 (15) | 2 (10) | 7.233 | 5.375 |
| **3** | 1 | 3 (0.9) | 3 (25) | 3 (25) | 4.149 | 3.374 |
| **4** | 2 (20.05) | 1 | 2 | 3 | 6.685 | 6.519 |
| **5** | 2 | 2 | 3 | 1 | 5.759 | 5.718 |
| **6** | 2 | 3 | 1 | 2 | 15.20 | 10.17 |
| **7** | 3 (30.08) | 1 | 3 | 2 | 4.203 | 4.196 |
| **8** | 3 | 2 | 1 | 3 | 10.55 | 10.59 |
| **9** | 3 | 3 | 2 | 1 | 17.79 | 10.89 |

**Table S2** L_9_(3^4^) Taguchi design space used for optimizing first- and second-eluted compounds of omeprazole; the design space is intended for the 80-bar pressure restriction.

| **Trial** | **A**  **u [cm min^–1^]** | **B**  **V_inj_ → t_inj_ [cm^3^]** | **C**  **L [cm]** | **D**  **d_p_ [µm]** | **Results: Pr/L × 10^4^**  **[g (min × cm)^–1^]** | |
| --- | --- | --- | --- | --- | --- | --- |
|  |  |  |  |  | **Compound 1 (S)** | **Compound 2 (R)** |
| **1** | 1 (2.77) | 1 (0.3) | 1 (10) | 1 (5) | 2.673 | 2.660 |
| **2** | 1 | 2 (0.6) | 2 (15) | 2 (10) | 2.299 | 1.888 |
| **3** | 1 | 3 (0.9) | 3 (25) | 3 (25) | 1.310 | 1.178 |
| **4** | 2 (5.55) | 1 | 2 | 3 | 2.325 | 2.323 |
| **5** | 2 | 2 | 3 | 1 | 1.778 | 1.777 |
| **6** | 2 | 3 | 1 | 2 | 7.081 | 4.315 |
| **7** | 3 (8.32) | 1 | 3 | 2 | 1.360 | 1.359 |
| **8** | 3 | 2 | 1 | 3 | 8.821 | 5.370 |
| **9** | 3 | 3 | 2 | 1 | 7.251 | 4.576 |

**Table S3** L_9_(3^4^) Taguchi design space used for optimizing first- and second-eluted compounds of etiracetam; the design space is intended for the 200-bar pressure restriction.

| **Trial** | **A**  **u [cm min^–1^]** | **B**  **V_inj_ → t_inj_ [cm^3^]** | **C**  **L [cm]** | **D**  **d_p_ [µm]** | **Results: Pr/L × 10^3^**  **[g (min × cm)^–1^]** | |
| --- | --- | --- | --- | --- | --- | --- |
|  |  |  |  |  | **Compound 1 (R)** | **Compound 2 (S)** |
| **1** | 1 (8.3) | 1 (0.3) | 1 (10) | 1 (5) | 4.659 | 3.187 |
| **2** | 1 | 2 (0.6) | 2 (15) | 2 (10) | 3.576 | 2.458 |
| **3** | 1 | 3 (0.9) | 3 (25) | 3 (25) | 1.797 | 1.245 |
| **4** | 2 (16.7) | 1 | 2 | 3 | 2.226 | 2.137 |
| **5** | 2 | 2 | 3 | 1 | 4.134 | 2.943 |
| **6** | 2 | 3 | 1 | 2 | 5.015 | 3.815 |
| **7** | 3 (25.0) | 1 | 3 | 2 | 3.483 | 3.442 |
| **8** | 3 | 2 | 1 | 3 | 0.7537 | 2.058 |
| **9** | 3 | 3 | 2 | 1 | 5.362 | 3.992 |

**Table S4** L_9_(3^4^) Taguchi design space used for optimizing first- and second-eluted compounds of etiracetam; the design space is intended for the 80-bar pressure restriction.

| **Trial** | **A**  **u [cm min^–1^]** | **B**  **V_inj_ → t_inj_ [cm^3^]** | **C**  **L [cm]** | **D**  **d_p_ [µm]** | **Results: Pr/L × 10^4^**  **[g (min × cm)^–1^]** | |
| --- | --- | --- | --- | --- | --- | --- |
|  |  |  |  |  | **Compound 1 (R)** | **Compound 2 (S)** |
| **1** | 1 (1.6) | 1 (0.3) | 1 (10) | 1 (5) | 13.10 | 9.578 |
| **2** | 1 | 2 (0.6) | 2 (15) | 2 (10) | 8.278 | 5.974 |
| **3** | 1 | 3 (0.9) | 3 (25) | 3 (25) | 4.423 | 3.017 |
| **4** | 2 (3.2) | 1 | 2 | 3 | 8.994 | 7.097 |
| **5** | 2 | 2 | 3 | 1 | 1.059 | 9.208 |
| **6** | 2 | 3 | 1 | 2 | 1.689 | 11.65 |
| **7** | 3 (4.8) | 1 | 3 | 2 | 8.278 | 8.277 |
| **8** | 3 | 2 | 1 | 3 | 7.882 | 8.165 |
| **9** | 3 | 3 | 2 | 1 | 23.06 | 16.84 |
